# Exploring patient acceptance of research within complex oral and IV outpatient parenteral antimicrobial therapy (COpAT) networks

**DOI:** 10.1093/jacamr/dlac087

**Published:** 2022-08-23

**Authors:** Timothy M Rawson, Theresa Eigo, Richard Wilson, Fran Husson, Rishi Dhillon, Owen Seddon, Alison Holmes, Mark Gilchrist

**Affiliations:** National Institute for Health Research Health Protection Research Unit in Healthcare Associated Infections and Antimicrobial Resistance, Imperial College London, Hammersmith Campus, Du Cane Road, London W12 0NN, UK; Centre for Antimicrobial Optimisation, Hammersmith Hospital, Imperial College London, Du Cane Road, London, W12 0NN, UK; Imperial College Healthcare NHS Trust, Hammersmith Hospital, Du Cane Road, London W12 0HS, UK; National Institute for Health Research Health Protection Research Unit in Healthcare Associated Infections and Antimicrobial Resistance, Imperial College London, Hammersmith Campus, Du Cane Road, London W12 0NN, UK; Centre for Antimicrobial Optimisation, Hammersmith Hospital, Imperial College London, Du Cane Road, London, W12 0NN, UK; Imperial College Healthcare NHS Trust, Hammersmith Hospital, Du Cane Road, London W12 0HS, UK; National Institute for Health Research Health Protection Research Unit in Healthcare Associated Infections and Antimicrobial Resistance, Imperial College London, Hammersmith Campus, Du Cane Road, London W12 0NN, UK; Public Health Wales Microbiology, University Hospital Wales, Heath Park, Cardiff, CF14 4XW, UK; Public Health Wales Microbiology, University Hospital Wales, Heath Park, Cardiff, CF14 4XW, UK; National Institute for Health Research Health Protection Research Unit in Healthcare Associated Infections and Antimicrobial Resistance, Imperial College London, Hammersmith Campus, Du Cane Road, London W12 0NN, UK; Centre for Antimicrobial Optimisation, Hammersmith Hospital, Imperial College London, Du Cane Road, London, W12 0NN, UK; National Institute for Health Research Health Protection Research Unit in Healthcare Associated Infections and Antimicrobial Resistance, Imperial College London, Hammersmith Campus, Du Cane Road, London W12 0NN, UK; Centre for Antimicrobial Optimisation, Hammersmith Hospital, Imperial College London, Du Cane Road, London, W12 0NN, UK; Imperial College Healthcare NHS Trust, Hammersmith Hospital, Du Cane Road, London W12 0HS, UK

Outpatient parenteral antimicrobial therapy (OPAT) is a safe and cost-effective means of delivering antimicrobial therapy for a number of infections.^1^ Review of the BSAC National OPAT Registry (NORS) between 2015 and 2019 demonstrated over 90% success from 27 841 treatment episodes.^1^ In addition to clinical outcome, patient-reported outcomes and satisfaction are superior with OPAT as patients are able to complete therapy from home rather than remaining in hospital for the total duration of treatment.^2^

OPAT requires multidisciplinary involvement and patients should have dedicated clinical management plans considering all aspects of their care. Pharmacokinetic (PK) challenges such as drug interactions, drug stability and vascular health issues can complicate therapy.^3^ Furthermore, self or caregiver administration can be a challenge.^4,5^

In recent years, evidence has emerged supporting the treatment of certain historic OPAT conditions with oral therapy. This avoids the need for an IV line, but still requires a dedicated clinical management plan for patients. To support this shift in delivery of care, complex outpatient antibiotic therapy (COpAT) services have been developed.^6^

The success of oral versus IV antimicrobial therapy relies on an ability to obtain optimal drug exposure, or antimicrobial PK, to achieve a required response for the specific infection. The effectiveness of oral treatment, pharmacodynamics (PD), is influenced by patient and organism factors including immune status, site of infection and MIC.^7^

OPAT networks within the UK lend themselves to large scale, multicentre clinical research and could support development of population-specific evidence around COpAT.^1^ However, the acceptability of clinical research to patients within this setting has yet to be fully assessed.

We undertook a survey of patients who were being actively being treated in two UK OPAT centres. A 10-question survey (available as Supplementary data at *JAC-AMR* Online) was developed and piloted on two independent researchers prior to its distribution. This pilot survey aimed to explore patient preferences for treatment approaches, in terms of follow-up monitoring and route of antimicrobial therapy and their acceptance of participation in clinical research as part of OPAT. This study was registered as a service evaluation with the local institutions.

Between February and April 2022, 26 patients attending weekly OPAT clinics at two OPAT centres within the UK were invited to complete the 10-question survey. Results were collated and summarized descriptively. Table 1 summarizes patient responses collected as part of the survey. Common indications for OPAT were skin and soft tissue (9/26; 35%), bone and joint (4/26; 15%) and intra-abdominal infections (3/26; 12%). Most patients were receiving IV antimicrobials (24/26; 92%) once a day (17/26; 65%) and attending their local OPAT infusion centre daily for therapy (14/26; 54%). The remaining 12/26 (46%) of patients received antimicrobials in the home environment administered by a district nurse (5/26; 19%), a family member (3/26; 12%) or via self-administration (4/26; 15%). Overall, patients reported that OPAT had only a mild impact on their daily activities (median score 3/10 where 1 = no impact and 10 = significant impact on daily activities).

**Table 1.** Summary of patient survey results from two OPAT centres in the UK

Description	Result (*n *= 26)
Diagnosis, *n* (%)	
Skin and soft tissue	9 (35)
Bone and joint	4 (15)
Urinary tract	2 (8)
Intra-abdominal	3 (12)
Other	8 (31)
Route of administration, *n* (%)	
IV	21 (81)
PO	2 (8)
IV/PO	3 (13)
Antibiotic frequency, *n* (%)	
Once a day	17 (65)
Twice a day	8 (31)
Other^a^	1 (4)
Administration, *n* (%)	
OPAT infusion centre	14 (54)
District nurses at home	5 (19)
Family member at home	3 (12)
Self-administration at home	4 (15)
Impact on daily life, 1–10 (1 minimal impact, 10 significant impact), median (range)	3 (1–10)
Preferred treatment, rank from 1–6 (1 preferred), median (IQR)	
Once weekly injection	3 (1–5)
Oral with telephone follow-up	2 (1–6)
Oral with weekly clinic	2 (2–3)
IV with weekly clinic	3 (1–6)
Oral, staying in hospital	5 (5–5)
IV, staying in hospital	6 (1–6)
Willing to participate in research on OPAT, *n* (%)	
No	8 (31)
Yes	11 (42)
Would consider with more information	5 (19)
Consider oral treatment compared to IV as part of a clinical trial, *n* (%)	
No	6 (23)
Yes	14 (54)
Would consider with more information	6 (23)

PO, oral.

a Four times a day oral dosing.

Patients were asked to rank options on their preferred approach to treatment from 1 (preferred) to 6 (least preferred). The most popular treatment approaches (median [IQR]) were oral therapy with weekly review in clinic (2 [2–3]), oral therapy with weekly telephone follow-up (2 [1–6]) and once weekly antibiotic injections (3 [1–5]). Twenty of 26 (77%) patients reported that they would consider taking part in a clinical research trial comparing oral to IV treatments as part of OPAT if offered. Of these, 6/20 (30%) would require more information prior to agreeing. Thematic analysis of free text comments identified common emerging themes that participants reported would help support their decisions to participate. These were presentation of data on the likely success of the oral antibiotic compared with IV, evidence supporting the treatment’s safety, and the additional impact on time that participating in clinical research would involve.

In conclusion, with the ongoing development of COpAT services there is a requirement to ensure that population-specific evidence is generated to support optimal treatment and approaches to deliver it. The OPAT/COpAT networks in the UK provide a vehicle to deliver large-scale, multicentre, clinical research trials. To deliver trials research through these networks, it is important to understand and address patient perspectives and requirements. This initial survey provides preliminary data from which larger-scale patient involvement and engagement activities can be developed to support the appropriate design, implementation and evaluation of clinical research trials in the OPAT/COpAT setting.

## Supplementary Material

dlac087_Supplementary_Data

## Supplementary data

The survey is available as Supplementary data at *JAC-AMR* Online.
