# Supplementary material for: Exploring patient acceptance of research within complex oral and IV outpatient parenteral antimicrobial therapy (COpAT) networks
Source: JAC Antimicrob Resist. 2022 Aug 23;4(4):dlac087. doi: 10.1093/jacamr/dlac087 (PMC9397122; doi:10.1093/jacamr/dlac087)
Supplement: dlac087_Supplementary_Data [file dlac087_supplementary_data.docx]

**Exploring patient experience of Outpatient Parenteral Antimicrobial Therapy (OPAT) and view on engagement with clinical research**

**Supplementary data**

Thank you for taking the time to complete this short survey. You have been asked to take part as you are currently receiving antibiotic treatment via the Outpatient Parenteral Antimicrobial Therapy (OPAT) service at Imperial College Healthcare NHS Trust. This survey will help us understand your experience of receiving antibiotics through the service. It will also help us understand the acceptability and potential challenges of offering future OPAT patients the opportunity to participate in clinical research trials.

If you need any help completing this survey, please ask a member of staff.

If you are happy to be contacted at a later date to discuss your answers to the survey in more detail, please let a member of the OPAT team know when you hand the survey in.

1. In your own words, what infection are you receiving antibiotics for?

**__________________________________________________________________**

2. How many times a day do you receive antibiotics?

Once a day

Twice a day

Three times a day

3. I take my antibiotics:

By mouth

Through a dedicated line

4. Who administers your antibiotics at home?

District Nurse

Family member

I do it myself

I go to the hospital / infusion centre

5. On the following scale, what impact does receiving your current antibiotics have on your day to day life (1 = no disruption at all, 10 = very disruptive to daily life).

| **No disruption** | **1** | **2** | **3** | **4** | **5** | **6** | **7** | **8** | **9** | **10** | **Very disruptive** |
| --- | --- | --- | --- | --- | --- | --- | --- | --- | --- | --- | --- |

We are really interested to hear your comments – please let us know any comments you have : ____________________________________________________________________________________________________________________________________________________________________________________________________________________________________________________________________________

6. If different options for treatment for your infection were available, please rank your order of preference (1 = best, 6= worse)

| **Treatment options:** | **Rank** |
| --- | --- |
| Once weekly antibiotic injection at the clinic |  |
| Daily oral antibiotics at home with telephone follow up |  |
| Daily oral antibiotics at home with follow up in clinic weekly |  |
| Daily intravenous antibiotics at home with follow up in clinic weekly |  |
| Daily oral antibiotics in hospital until completed treatment |  |
| Daily intravenous antibiotics in hospital until completed treatment |  |

Please comment: ____________________________________________________________________________________________________________________________________________________________________________________________________________________________________________________________________________

7. During your time on OPAT, would you have considered participating in a clinical research trial relating to your antibiotic treatment if this were offered to you?

Yes

No

Unsure

Please comment on your reason for this answer: ____________________________________________________________________________________________________________________________________________________________________________________________________________________________________________________________________________

8: If you were offered an oral antibiotic treatment instead of the standard intravenous option as part of a clinical research trial would you have considered participating?

Yes

No

Unsure

Please comment on your reason for this answer: ____________________________________________________________________________________________________________________________________________________________________________________________________________________________________________________________________________

Thank you for taking the time to complete this survey!
